# Prevalence and Load of the Campylobacter Genus in Infants and Associated Household Contacts in Rural Eastern Ethiopia: a Longitudinal Study from the Campylobacter Genomics and Environmental Enteric Dysfunction (CAGED) Project

**DOI:** 10.1128/aem.00424-23

**Published:** 2023-06-13

**Authors:** Loïc Deblais, Amanda Ojeda, Mussie Brhane, Bahar Mummed, Kedir A. Hassen, Belisa Usmael Ahmedo, Yenenesh Demisie Weldesenbet, Jafer Kedir Amin, Ibsa Abdusemed Ahmed, Ibsa Aliyi Usmane, Efrah Ali Yusuf, Abadir Jemal Seran, Fayo I. Abrahim, Halengo T. Game, Ballo A. Mummed, Meri M. Usmail, Kunuza Adem Umer, Mawardi M. Dawid, Wondwossen Gebreyes, Nigel French, Jemal Yousuf Hassen, Kedir Teji Roba, Abdulmuen Mohammed, Getnet Yimer, Cyrus Saleem, Dehao Chen, Nitya Singh, Mark J. Manary, Sarah L. McKune, Arie H. Havelaar, Gireesh Rajashekara

**Affiliations:** a The Ohio State University, Columbus, Ohio, USA; b University of Florida, Gainesville, Florida, USA; c Haramaya University, Dire Dawa, Ethiopia; d Massey University, Palmerston North, New Zealand; e Global One Health Initiative, The Ohio State University, Addis Ababa, Ethiopia; f Washington University, St. Louis, Missouri, USA; Centers for Disease Control and Prevention

**Keywords:** *Campylobacter*, real-time PCR, longitudinal study, eastern Ethiopia, livestock feces, human stool

## Abstract

In our previous cross-sectional study, multiple species of Campylobacter were detected (88%) in stool samples from children (12 to 14 months of age) in rural eastern Ethiopia. This study assessed the temporal fecal carriage of Campylobacter in infants and identified putative reservoirs associated with these infections in infants from the same region. The prevalence and load of Campylobacter were determined using genus-specific real-time PCR. Stool samples from 106 infants (*n* = 1,073) were collected monthly from birth until 376 days of age (DOA). Human stool samples (mothers and siblings), livestock feces (cattle, chickens, goats, and sheep), and environmental samples (soil and drinking water) from the 106 households were collected twice per household (*n* = 1,644). Campylobacter was most prevalent in livestock feces (goats, 99%; sheep, 98%; cattle, 99%; chickens, 93%), followed by human stool samples (siblings, 91%; mothers, 83%; infants, 64%) and environmental samples (soil, 58%; drinking water, 43%). The prevalence of Campylobacter in infant stool samples significantly increased with age, from 30% at 27 DOA to 89% at 360 DOA (1% increase/day in the odds of being colonized) (*P* < 0.001). The Campylobacter load increased linearly (*P* < 0.001) with age from 2.95 logs at 25 DOA to 4.13 logs at 360 DOA. Within a household, the Campylobacter load in infant stool samples was positively correlated with the load in mother stool samples (*r*^2^ = 0.18) and soil collected inside the house (*r*^2^ = 0.36), which were in turn both correlated with Campylobacter loads in chicken and cattle feces (0.60 < *r*^2^ < 0.63) (*P* < 0.01). In conclusion, a high proportion of infants are infected with Campylobacter in eastern Ethiopia, and contact with the mother and contaminated soil may be associated with early infections.

**IMPORTANCE** A high Campylobacter prevalence during early childhood has been associated with environmental enteric dysfunction (EED) and stunting, especially in low-resource settings. Our previous study demonstrated that Campylobacter was frequently found (88%) in children from eastern Ethiopia; however, little is known about potential Campylobacter reservoirs and transmission pathways leading to infection of infants by Campylobacter during early growth. In the longitudinal study presented here, Campylobacter was frequently detected in infants within the 106 surveyed households from eastern Ethiopia, and the prevalence was age dependent. Furthermore, preliminary analyses highlighted the potential role of the mother, soil, and livestock in the transmission of Campylobacter to the infant. Further work will explore the species and genetic composition of Campylobacter in infants and putative reservoirs using PCR and whole-genome and metagenomic sequencing. The findings from these studies can lead to the development of interventions to minimize the risk of transmission of Campylobacter to infants and, potentially, EED and stunting.

## INTRODUCTION

The World Health Organization (WHO) classifies infectious diarrhea caused by enteric pathogens as one of the most prevalent causes of death in infants under 5 years of age (over 500,000 deaths annually) (1). This alarming phenomenon is especially true in low-income countries, where young children (<24 months of age) experience an average of 3 episodes of diarrhea annually (2, 3). Diarrheal diseases are often associated with increased gut inflammation and intestinal permeability, ultimately leading to a “leaky” gut (4, 5). As a consequence, the infant is unable or has a limited capacity to absorb nutrients from breast milk, which are essential for the proper development of the infant (6, 7). Therefore, diarrheal diseases have been well recognized as significant determinants of malnutrition, stunting, and cognitive deficits (8–10). In Ethiopia, diarrhea is the second most prevalent cause of mortality in infants under the age of 5 years, with an overall 12% national prevalence of childhood diarrheal cases (11, 12). Despite remarkable efforts to reduce infant mortality over the past decades, diarrheal diseases are still a recurrent problem (13). Several studies have attempted to explore the determinants contributing to diarrheal disease in Ethiopia (11, 14–19); however, it is a complex public health threat modulated by various socioeconomic, environmental, and behavioral factors. For example, dysbiosis is also associated with asymptomatic infections (12, 20). Therefore, broad-scale longitudinal studies powered by multidisciplinary approaches are needed to address this global health concern (11).

The guts of “unhealthy” infants prone to dysbiosis are frequently characterized by a predominance of enteropathogenic bacteria (i.e., Enterococcus faecalis, Clostridium difficile, and Campylobacter spp.) (21, 22). Campylobacter is a Gram-negative bacterium with diverse human and animal reservoirs and is a leading cause of diarrheal disease worldwide (23). In low-income countries, children under the age of 2 years are particularly susceptible to Campylobacter-induced diarrhea (24). Several cross-sectional and longitudinal studies have found that both symptomatic and nonsymptomatic Campylobacter infections are associated with linear growth impairment (12, 23, 25–27). A recent study in Malawian children also showed that asymptomatic Campylobacter infection was indirectly associated with lower length-for-age Z scores (LAZs) at 24 months of age, with 69.6% of asymptomatic children being positive for Campylobacter (28).

In 2018, our team conducted a cross-sectional study in rural eastern Ethiopia as part of the CAGED (Campylobacter Genomics and Environmental Enteric Dysfunction) project. Using an advanced metagenomic sequencing approach (meta-total RNA sequencing), we demonstrated that various species of the Campylobacter genus were detected in 88% (*n* = 88/100) of the stool samples collected from children between 360 and 498 days of age (DOA). In addition to the well-known thermotolerant species Campylobacter jejuni and C. coli, we detected an unidentified nonthermotolerant species (20). Similar trends were reported in a previous study in Hawassa, Ethiopia (25). Therefore, early infection of infants by Campylobacter seems to be predominant in Ethiopia.

Based on these findings, a longitudinal study was conducted with 106 selected households between December 2020 and June 2022 in the same region in rural eastern Ethiopia. The overall objectives and study design were reported previously (29). The objectives of this specific study were to assess the patterns of the temporal colonization of infants with Campylobacter and to identify potential reservoirs associated with Campylobacter infections in infants from rural eastern Ethiopian households. The prevalence and load of Campylobacter in human stool samples, animal feces, and environmental samples were determined using genus-specific real-time PCR.

## RESULTS

### The prevalence and load of Campylobacter are high in households in rural eastern Ethiopia.

**FIG 1:**
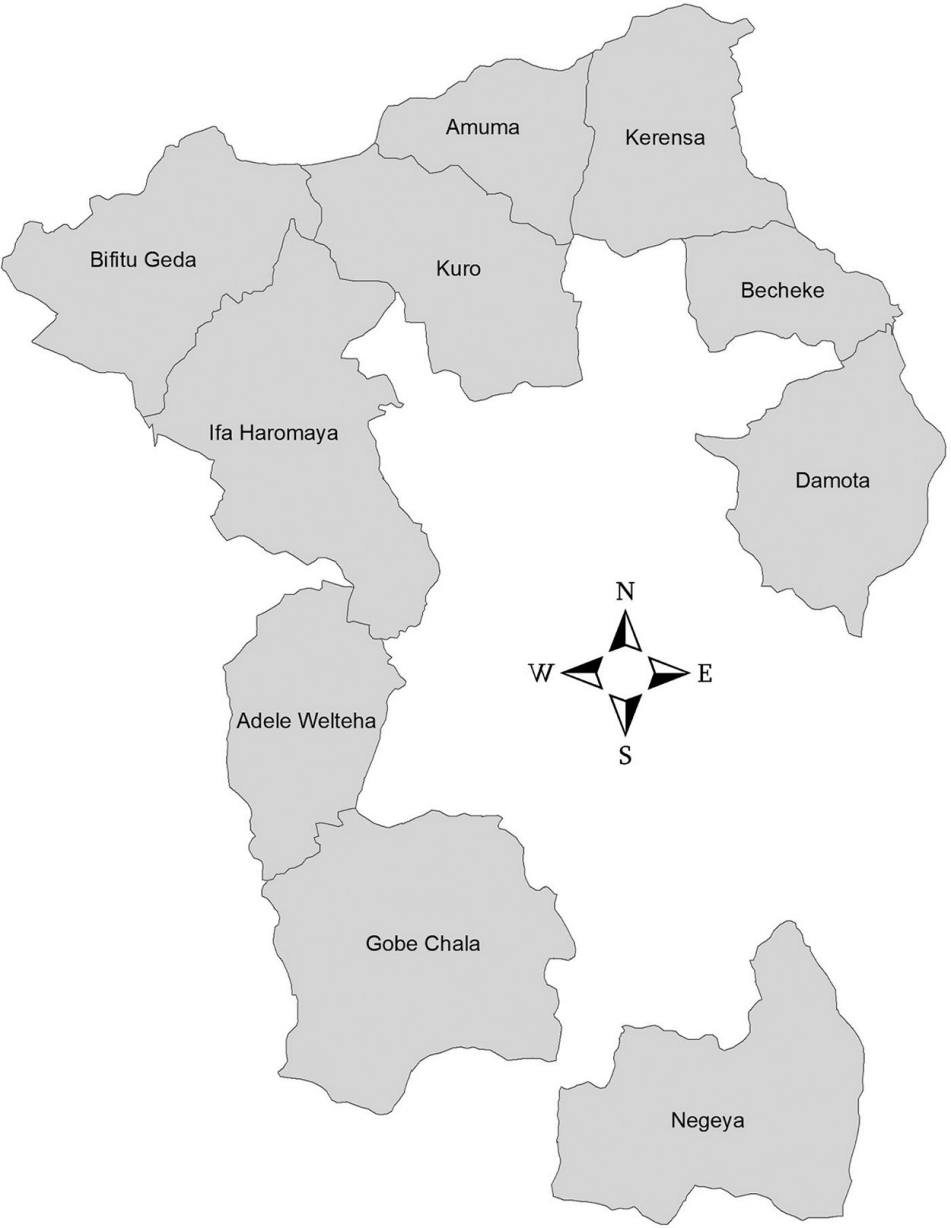
Geographic locations of the households selected for the CAGED longitudinal study. A total of 106 households located within 10 kebeles (villages) from the Haramaya District in eastern Ethiopia were selected. Human stool samples (infants, siblings, and mothers), livestock feces (cattle, chickens, goats, and sheep), and environmental samples (drinking water and soil) were collected between December 2020 and June 2022. Additional details about the population size for each kebele and sample type are included in Table S1 in the supplemental material (29).

A total of 2,717 samples were collected between December 2020 and June 2022 across the 106 selected households located within 10 kebeles (synonymous with villages) (approximately 143 ± 75 samples collected per month) (Fig. 1; see also Table S1 in the supplemental material) in the Haramaya woreda, Eastern Hararghe Zone, Ethiopia. An average of 272 (minimum/maximum, 209/331) samples were collected per kebele. Each kebele contained approximately 11 households (minimum/maximum, 8/12), and an average of 26 (minimum/maximum, 12/33) samples were collected per household. Samples were subdivided into different categories: human stool samples (1,073 from infants, 147 from siblings, and 136 from mothers), livestock feces (200 from cattle, 161 from chickens, 197 from sheep, and 199 from goats), and environmental samples (148 from drinking water and 456 from soil). Human samples were taken independently of the presence or absence of diarrhea.

**FIG 2:**
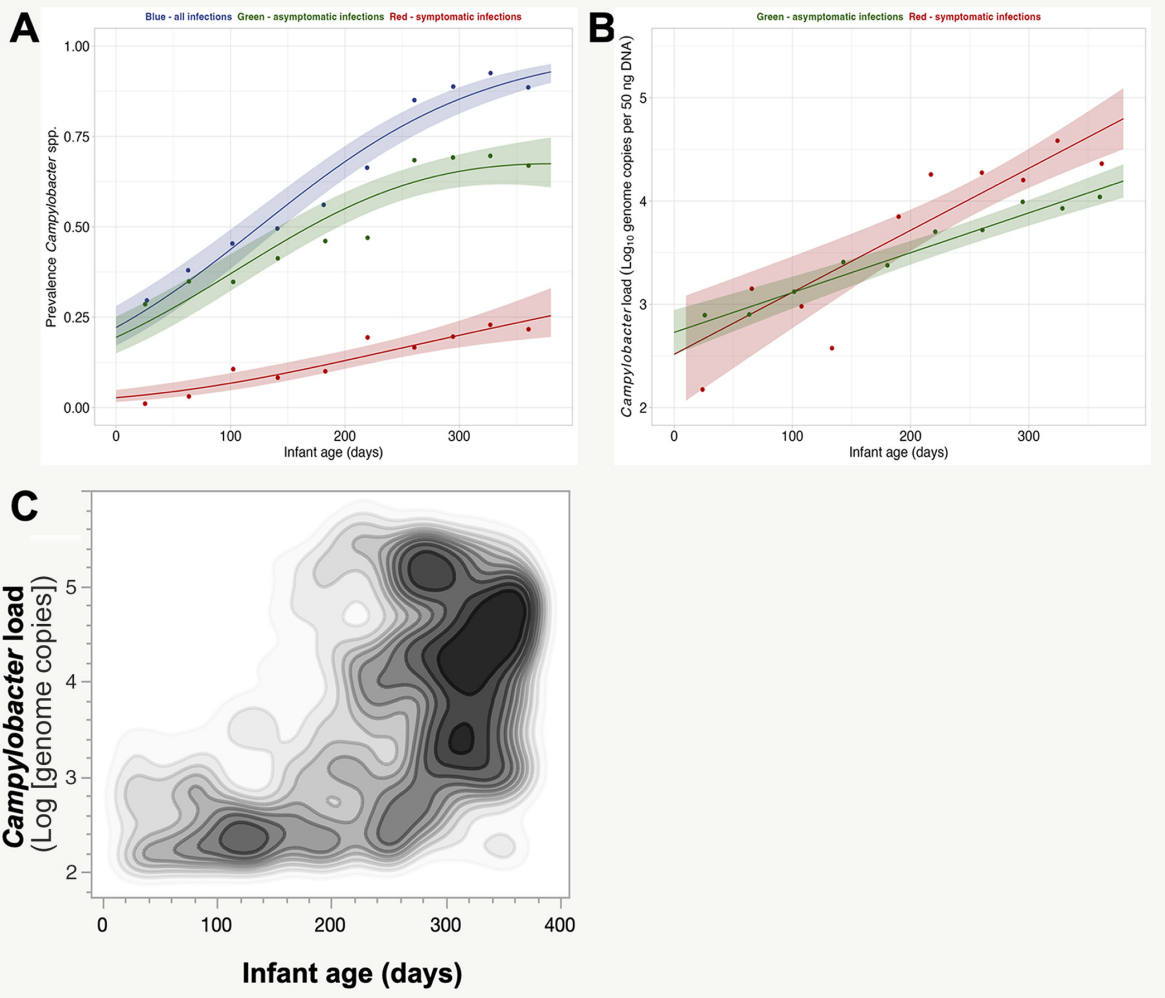
Prevalence and load of Campylobacter in infant stool samples over time. (A) Campylobacter prevalence in infant stool samples by age and the presence or absence of diarrhea. Points are prevalences by 4-week age group, lines are best-fitting logistic regression models of presence/absence as a function of age as a continuous variable, and shaded areas are 95% confidence intervals. (B) Campylobacter loads in positive infant stool samples over time. Points are average loads by 4-week age group, lines are best-fitting linear regression models of load as a function of age as a continuous variable, and shaded areas are 95% confidence intervals. (C) Campylobacter loads in positive infant stool samples as a function of age. Shades of gray are proportional to the numbers of stool samples harboring similar Campylobacter loads for the indicated infant age.

The prevalence of Campylobacter in the human stool samples, animal feces, soil samples, and drinking water samples was determined based on the threshold cycle (*C_T_*) values obtained for the samples versus the no-DNA controls (water control). These *C_T_* values provided an estimate of the background noise (fluorescence signal obtained by nonspecific amplification), which was then used to determine whether a sample was positive or negative for Campylobacter. The data presented below were generated using the following criteria. A cutoff *C_T_* value of 35 was used for the detection of Campylobacter in the samples (average *C_T_* value for nuclease-free water − 2.5 × standard deviation). A cutoff *C_T_* value of 29 was used for the detection of Campylobacter in the soil samples due to the low *C_T_* values obtained from soil samples free of Campylobacter compared to the water controls. *C_T_* values from positive samples were converted into Campylobacter genome copies per 50 ng of DNA tested, as described in “Detection of Campylobacter using TaqMan real-time PCR” in Materials and Methods. Overall, 73.6% (2,001/2,717) of the samples tested positive for Campylobacter at the genus level (Fig. 2A and Table S2). Significant differences in Campylobacter prevalences were observed between kebeles (Table S2). Overall, Adele Welteha harbored a significantly higher proportion of Campylobacter-positive samples (81% [95% confidence interval {CI}, 76% to 86%]) than the overall Campylobacter-positive samples in this study (74% [95% CI, 72% to 75%]) (*P* = 0.002). Furthermore, the Campylobacter prevalence was lower in the drinking water collected from Damota (5%) than the prevalence across all kebeles (43.2%) (*P* < 0.05). Drinking water from Adele Welteha harbored a Campylobacter prevalence of 25%, but the sample size was too small for the results to be significant (*n* = 4). The Campylobacter prevalence was lower in the mothers’ stool samples collected from Gobe Chala (30%) than the prevalence across all kebeles (83.1%) (*P* < 0.05). No significant differences in Campylobacter loads by sample type were detected in the positive samples between kebeles (approximately 3.66 [95% CI, 3.63 to 3.70] genome copies per 50 ng of DNA).

### The prevalence and load of Campylobacter in infants from rural eastern Ethiopia increase with age.

A total of 1,073 infant stool samples were analyzed from the 106 infants included in this study. Fifty-two percent (55/106) were male infants, and 48% (51/106) were female infants. Stool samples were collected when infants were between 7 and 376 days of age. Overall, Campylobacter was detected at least once in all infants, and 63.8% (95% CI, 60.9% to 66.7%) of the stool samples were positive for Campylobacter. The average Campylobacter load in positive infant stool samples was 3.67 (95% CI, 3.59 to 3.75) log_10_ genome copies per 50 ng of DNA. Campylobacter prevalences (60% for males and 63% for females) and average loads (3.68 [95% CI, 3.57 to 3.80] and 3.77 [95% CI, 3.66 to 3.90] log_10_ genome copies for males and females, respectively) were not different between sexes. The prevalence of Campylobacter in infant stool samples significantly increased with age, from 30% at 27 days of age to 89% at 360 days of age (1% increase per day in the odds of being colonized) (*P* < 0.001) (Fig. 2A). The prevalence of diarrhea increased from 2.4% at 27 days to 25% at 360 days of age. The proportion of infants with Campylobacter in their stool and having diarrhea as predicted by a logistic regression model was 33% on average, decreasing from 56% after birth to 29% at 380 days of age. The Campylobacter load in colonized infants increased linearly (*P* < 0.001) with age from 2.95 log_10_ genome copies at 25 days of age to 4.13 log_10_ genome copies at 360 days (Fig. 2B and C). In symptomatic children, the increase was higher (increase of 0.006 per day) than in asymptomatic children (increase of 0.004 per day), leading to a significantly higher load in symptomatic children after 220 days. Details about the Campylobacter prevalence data by individual infants over time are described in Fig. S1. Overall, the population was divided into three clusters. The first cluster was composed of infants (*n* = 26) from whom Campylobacter was repeatedly detected in the stool samples collected during the first 150 days of age. The second cluster was composed of infants (*n* = 39) from whom Campylobacter was repeatedly detected in the stool samples collected after the first 150 days of age. The third cluster was composed of infants (*n* = 41) from whom Campylobacter was inconsistently detected in the stool samples collected during the longitudinal study.

A weak correlation (*r*^2^ = 0.14; *P* < 0.001) was detected between the load of Campylobacter in the infant stool samples and infant age because the Campylobacter load for a designated infant was highly variable over time. The prevalence and load of Campylobacter in infant stool samples were closely associated with infant age. No association could be detected based on the season.

### Prevalence and load of Campylobacter in the putative environmental sources surrounding the infant.

**FIG 3:**
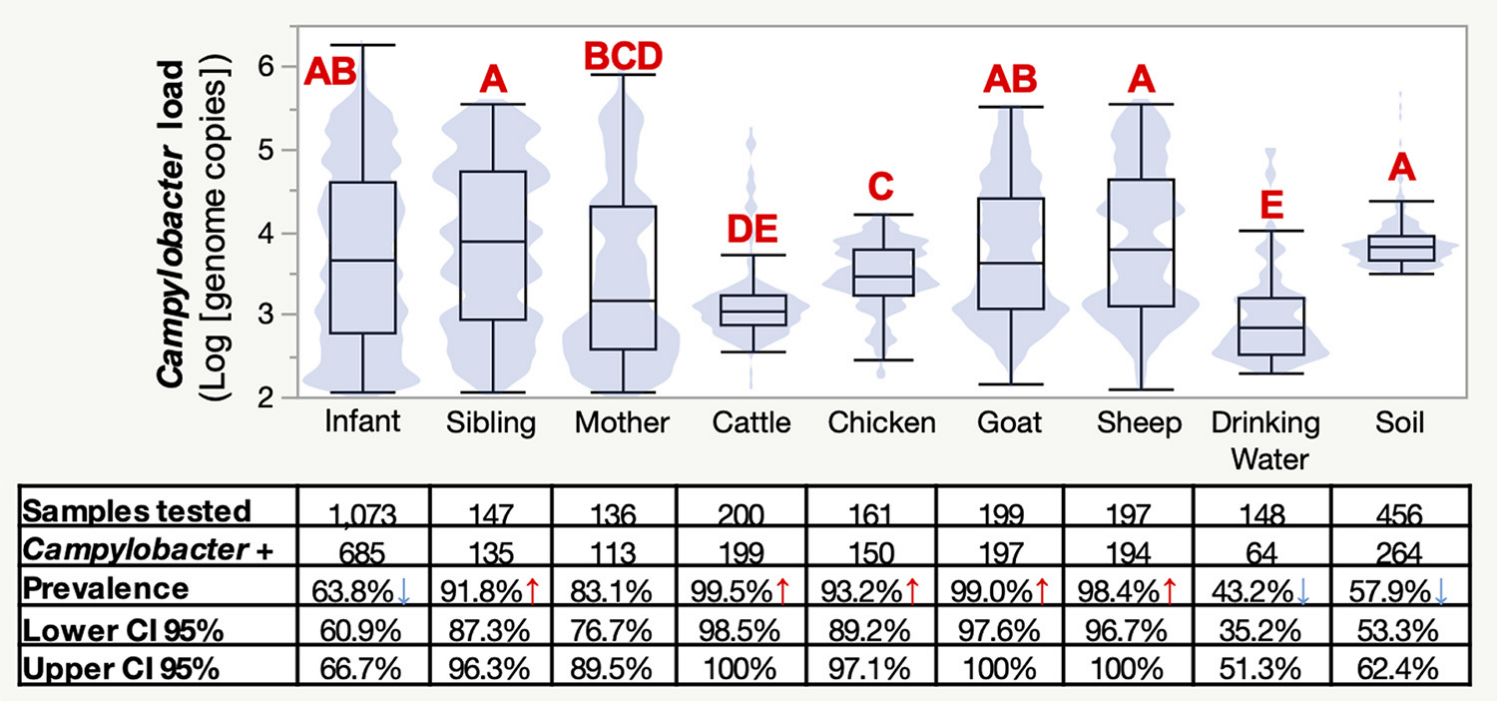
Prevalence and abundance of Campylobacter in the 106 selected households. The boxplot shows the Campylobacter loads in positive samples (Campylobacter genome copies per 50 ng of DNA tested) per sample type. Blue shading indicates the distribution of the Campylobacter load data within the selected sample type. Letters (A to E) indicate statistical differences (*P* < 0.05). The table at the bottom displays Campylobacter prevalences (with lower and upper 95% confidence intervals [CI]) for the associated samples. Red and blue arrows indicate whether the prevalence is significantly higher or lower, respectively, than the Campylobacter prevalence across all of the samples (73.6%) (*P* < 0.05). Campylobacter + indicates the number of Campylobacter-positive samples.

Overall, Campylobacter was most commonly found in livestock feces (97.7% [95% CI, 96.7% to 98.8%]), followed by human stool samples (68.8% [95% CI, 66.3% to 71.3%]) and environmental samples (54.3% [95% CI, 50.3% to 58.3%]) (*P* < 0.001) (Fig. 3). On the other hand, Campylobacter loads were similar across the three sample types (3.66 [95% CI, 3.59 to 3.73] log_10_ genome copies for human stool samples, 3.65 [95% CI, 3.59 to 3.72] log_10_ genome copies for environmental samples, and 3.58 [95% CI, 3.52 to 3.63] log_10_ genome copies for livestock feces) (Fig. 3).

Among the household members (infants, siblings, and mothers) studied in the 106 households, Campylobacter was more frequently detected in mother and sibling stool samples (83.1% [95% CI, 76.7% to 89.5%] and 91.8% [95% CI, 87.3% to 96.3%], respectively) than in the infant stool samples (63.8% [95% CI, 60.9% to 66.7%]) (*P* < 0.0001). A similar trend was observed for the bacterial load data (*P* < 0.04) (Fig. 3). Interestingly, the Campylobacter prevalence in mother stool samples increased in the months following giving birth; within the first 50 days after birth, 50% of the mother stool samples were positive for Campylobacter, while 90% were positive once the infant was over 100 days of age. However, we acknowledge that these data are preliminary, and mother stool samples collected before birth are required to support these observations.

Among the livestock (cattle, chickens, goats, and sheep) studied in the 106 selected households, the Campylobacter prevalence was highest among cattle (99.5% [95% CI, 98.5% to 100%]), goat (99.0% [95% CI, 97.6% to 100%]), and sheep (98.4% [95% CI, 96.7% to 100%]) feces compared to chicken feces (93.2% [95% CI, 89.2% to 97.1%]) (*P* < 0.05) (Fig. 3). Sheep and goat feces had higher Campylobacter loads (3.90 [95% CI, 3.77 to 4.02] and 3.78 [95% CI, 3.59 to 3.75] log_10_ genome copies, respectively) (*P* < 0.001), followed by chicken feces (3.46 [95% CI, 3.59 to 3.75] log_10_ genome copies) and cattle feces (3.16 [95% CI, 3.09 to 3.23] log_10_ genome copies) (*P* < 0.0006) (Fig. 3).

Among the drinking water and soil samples studied in the 106 selected households, Campylobacter was more frequently detected and more abundant in the soil samples (57.9% [95% CI, 53.3% to 62.4%] and 3.87 [95% CI, 3.83 to 3.91] log_10_ genome copies) than in the drinking water (43.2% [95% CI, 35.2% to 51.3%] and 2.78 [95% CI, 2.63 to 2.93] log_10_ genome copies) (*P* < 0.001) (Fig. 3). The Campylobacter load was higher in the soil collected during the rainy season (3.99 [95% CI, 3.88 to 4.10] log_10_ genome copies between June and August) than in the soil collected during the dry season (3.82 [95% CI, 3.79 to 3.85] log_10_ genome copies between October and May) (Fig. S2). Overall, the prevalence and load of Campylobacter in the soil were different based on the location selected within the household (inside versus outside the household) and whether humans have direct contact with dirt (e.g., soil covered with a carpet). Soil samples in direct contact with dust and dirt collected outside and inside the house were more likely to be positive for Campylobacter (73.7% [95% CI, 66.6% to 80.8%] and 71.7% [95% CI, 64.5% to 78.9%], respectively) and harbored higher loads (3.93 [95% CI, 3.86 to 4.01] and 3.87 [95% CI, 3.82 to 3.91] log_10_ genome copies, respectively) than soil samples collected inside the house where the soil was covered with a carpet (28.3% [95% CI, 21% to 35.5%] and 3.71 [95% CI, 3.66 to 3.75] log_10_ genome copies, respectively). The prevalence and load of Campylobacter in drinking water collected varied between kebeles (between 5% and 50% and between 2.71 and 4.04 log_10_ genome copies per 50 ng of DNA, respectively).

### Cooccurrence of Campylobacter within households in rural eastern Ethiopia.

**FIG 4:**
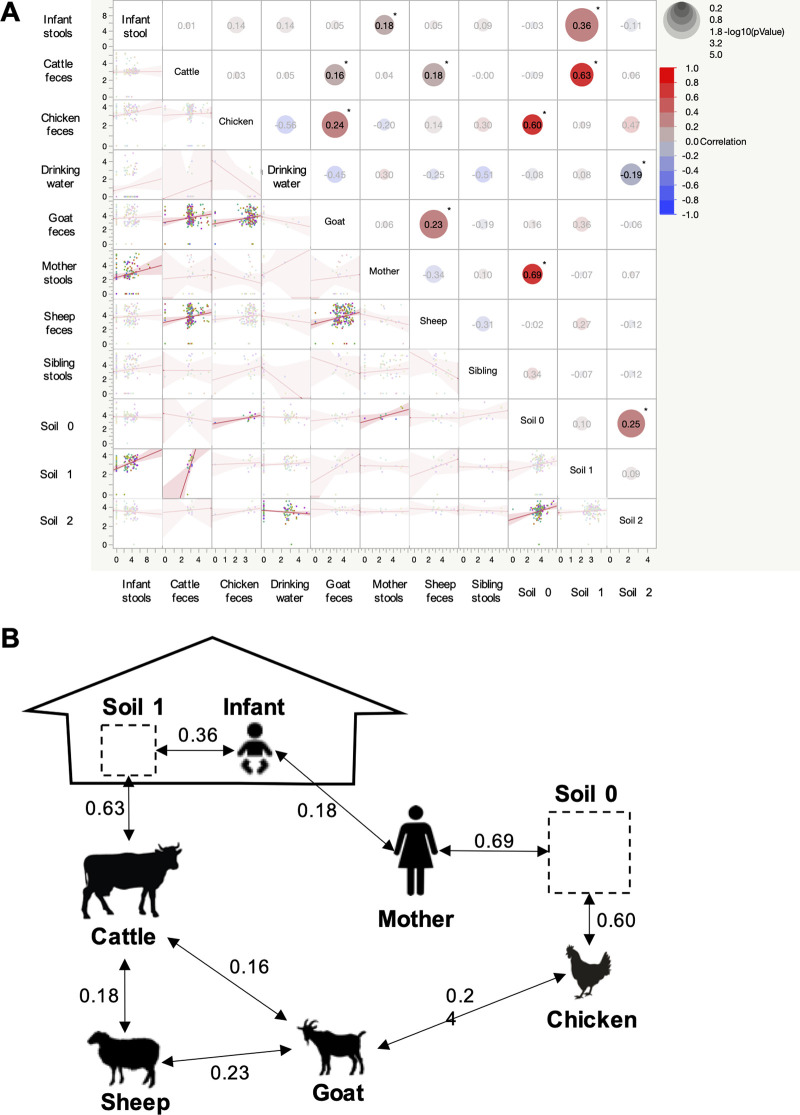
Correlation of Campylobacter loads between sample types. (A) Multivariate analysis. Campylobacter load data were compared between sample types within the indicated households. The sizes of the circles are proportional to the significance of the findings (*P* value). The color of the circle is associated with the correlation trend (positive in red and negative in blue) and intensity (*r*^2^ value). Asterisks indicate a significant correlation between the indicated sample types (*P* < 0.05). Soil 0 indicates soil samples collected outside the house, Soil 1 indicates soil samples collected inside the house where the soil was covered with a carpet or plastic sheet, and Soil 2 indicates soil samples collected inside the house where the soil was not covered and likely was close to the exit door. (B) Visual representation of the potential pathways for the transmission of Campylobacter to infants within a household in eastern Ethiopia. Based on the significant correlations obtained in panel A, potential links (arrows and associated *r*^2^ values) were identified between the different host/environment samples studied based on the Campylobacter load data generated using genus-specific real-time PCR.

A multiple pairwise correlation analysis was conducted to decipher correlations in Campylobacter loads between the human stool samples, animal feces, soil samples, and drinking water samples (*n* = 9 sample types collected per household) collected across the 106 selected household over time (*n* = 2,717 samples collected) (Fig. 4A). Out of the 55 combinations tested, 11 combinations had significant correlations based on the Campylobacter load data (*P* < 0.05). Interestingly, all of them but one displayed positive correlations (0.16 < *r*^2^ < 0.69). Several correlations occurred between livestock fecal samples (cattle, chickens, goats, and sheep) (*n* = 4). The Campylobacter load in goat feces was positively correlated with the loads in cattle (*r*^2^ = 0.16) and chicken (*r*^2^ = 0.24) feces. Similarly, the Campylobacter load in sheep feces was positively correlated with the loads in the cattle (*r*^2^ = 0.18) and goat (*r*^2^ = 0.23) feces. Correlations between human stool samples/livestock feces and soil samples collected at different locations within the household were also frequently detected (*n* = 5). The Campylobacter load in covered soil inside the home was positively correlated with the load in cattle feces (*r*^2^ = 0.63), and the load in soil outside the home was positively correlated with the load in infant feces (*r*^2^ = 0.60). No correlation was observed for uncovered soil in the home. The load of Campylobacter in the mother and infant stool samples was positively correlated with the load in soil samples collected outside the household (*r*^2^ = 0.69 and 0.36, respectively). The load of Campylobacter in the mother stool samples also correlated with the load in the child stool samples (*r*^2^ = 0.18). No correlation was observed for sibling stool samples. Overall, infection of infants by Campylobacter seems to be correlated with two distinct pathways, both involving soil and livestock feces (Fig. 4B). Interestingly, the load of Campylobacter in drinking water was negatively correlated with the load in soil samples (*r*^2^ = −0.19). Additional details about the unique correlations detected by kebele are described in Fig. S3.

Similarly, an association study was conducted to determine whether the prevalence of Campylobacter in specific sample types within the household could be linked to higher contamination risks for the infant. Overall, a market basket analysis identified only three strong associations that were detected between the prevalence of Campylobacter in the infant stool samples and the prevalence of Campylobacter in the livestock feces (cattle, goats, and sheep) (confidence of 95 to 98% and lift of 4.3 to 4.4) (Table 1). On the other hand, numerous strong associations (confidence of 95 to 98% and lift of 4.3 to 4.4) were detected between livestock species (*n* = 7/17) or between soil locations (*n* = 7/17) (Table 1).

**TABLE 1:** Campylobacter prevalence association study between sample types using market basket analysis^*a*^

Variable 1, if Campylobacter is detected in:	Variable 2, Campylobacter is likely to also be detected in:	Confidence level (%)	Lift
Soil 0	Soil 2	99	5.743
Soil 2	Soil 0	99	5.743
Soil 0, soil 1	Soil 2	99	5.741
Soil 1, soil 2	Soil 0	99	5.741
Soil 1	Soil 0	99	5.701
Soil 1	Soil 2	99	5.701
Soil 1	Soil 0, soil 2	98	5.699
Cattle, child	Goat	98	4.379
Child, sheep	Goat	97	4.336
Chicken, goat	Cattle	96	4.382
Chicken, goat, sheep	Cattle	96	4.371
Chicken, sheep	Cattle	96	4.346
Cattle, chicken, sheep	Goat	95	4.27
Chicken	Cattle	95	4.33
Cattle, chicken	Goat	95	4.253
Chicken, sheep	Goat	95	4.246
Child, goat	Sheep	95	4.313

a The confidence level indicates the likelihood that the prediction is true. Lift (ratio of the confidence to the expected confidence) indicates the likelihood (fold) that one sample type also contains Campylobacter (variable 2) if it is already detected in a set of sample types (variable 1) for the indicated household. Soil 0 indicates soil samples collected outside the house, soil 1 indicates soil samples collected inside the house where the soil was covered with a carpet or a plastic sheet, and soil 2 indicates soil samples collected inside the house where the soil was not covered and was likely close to the exit door.

## DISCUSSION

One of the objectives of our longitudinal study was to investigate the prevalence and load of Campylobacter in stool samples collected from 106 infants from eastern Ethiopia during the first 376 days after birth. To ensure that the data presented in this study are representative of the studied region, several selection criteria were used, which were described in great detail previously (29). Briefly, our study focused on 106 households distributed across 10 of the 36 rural kebeles from the Haramaya woreda (synonym for district) (29). This area was of special interest because the Haramaya woreda harbors a high rate of stunting (45.8%) compared to the national average (38%) (29, 30). A population of 106 households allowed the estimation of a 50% prevalence with a precision of 10% at 95% confidence and a power of 80%. Households were selected for this study if the family stayed in the Haramaya woreda during the study, the mother already lived in the woreda for at least 3 months during pregnancy, and the mother was over 16 years of age when giving birth. Infants were selected only if (i) their birth weight was above 2.5 kg, (ii) the infant or mother stayed in the hospital for <4 days after birth, and (iii) the infant had no visible congenital abnormality or known serious medical illness or enteropathy, diagnosed by a medical doctor (29).

Overall, our study demonstrated that Campylobacter was highly prevalent (63.8% positive stool samples across all ages) in the surveyed infants of Haramaya woreda between December 2020 and June 2022 using quantitative PCR (qPCR). In our previous formative research conducted in 2018, we found 88% positive stool samples using meta-total RNA sequencing (MeTRS) (20, 31). The difference in the Campylobacter prevalences observed between the two studies might be due to the age of the infants, although differences between analysis methods may also have contributed. During the formative study, stool samples were collected when the child was between 12 and 16 months of age (31), while during the longitudinal study, stool samples were collected on a monthly basis from birth until approximately 12 months of age. Our longitudinal study showed that the prevalence of Campylobacter in infant stool samples is positively correlated with the age of the infant (*r*^2^ = 0.94; *P* < 0.001). Older infants (>180 days of age; ca. 6 months of age) had a 6.8-times-higher odds of harboring Campylobacter, and the prevalence plateaued at 84.6% after 250 days of age (ca. 8 to 12 months of age), which concurs with our formative research findings (31). During our longitudinal study, Campylobacter was detected in at least 30% of the stool samples collected within the first 27 days of infant life. This finding may be explained by the pre- or neonatal transmission of Campylobacter from mother to infant, as has been reported for, e.g., Campylobacter curvus and C. fetus (32–35). Alternatively, early exposure from environmental sources might contribute to infection shortly after birth. More detailed analyses focusing on risk factors for exposure and assessing the species and genomic compositions of the Campylobacter population to understand colonization patterns are ongoing and will be communicated in our future publications (Chen et al., unpublished data; Li et al., unpublished data). We also observed that the Campylobacter burden in infant stool samples increased with age (ca. 1% increase per day in the odds of being colonized); this increase was associated with an increase in the prevalence (an increase of ca. 0.006 log_10_ genome copies per day in symptomatic infants versus an increase of 0.004 log_10_ genome copies per day in asymptomatic children). The Campylobacter prevalence results in our study are in concordance with those of other longitudinal studies conducted in low- and middle-income countries where the Campylobacter prevalence increased as the child became older (26, 27, 36, 37). For example, in South Africa, the highest prevalence of Campylobacter in infant stool samples was detected at between 8 and 11 months of age (18 to 19%) compared to younger (9 to 12% at between 0 and 4 months of age) and older (13 to 14% at between 15 and 24 months of age) age ranges. Similar trends were reported in the MAL-ED study in Tanzania (36). It is important to mention that an enzyme immunoassay, not a molecular approach (qPCR or sequencing), was used to detect Campylobacter in the South African study, which could explain the lower prevalence in the infant stool samples in the South African study than in our study (between 32 and 88% depending on infant age). Platt-Mills et al. also recommended the use of PCR-based approaches to detect Campylobacter due to their high sensitivity and specificity (38). Even though an overall increase in the Campylobacter load was observed in the positive stool samples collected over time at the population level (*n* = 106 infants surveyed in this study), the Campylobacter loads in infant stool samples did not consistently increase over time. We hypothesize that the rapid change in the gut microbiome during early life (21, 39) could significantly affect the ability of Campylobacter to colonize the infant gut (40–43). Additional Campylobacter infections may occur several times during early infant life. As reported in other studies, including ours, the infants may be in contact with a broad diversity of Campylobacter species from various reservoirs surrounding the infant at later ages (31, 44). Future efforts will focus on studying the Campylobacter composition in infant stool samples at the species level by using species-specific qPCR and conducting functional genomic analyses to study the virulome, the resistome, and other functional pathways in Campylobacter (45–48). In addition, the stool microbiome of the 106 infants surveyed until 12 months of age will be analyzed to decipher biomarkers associated with the persistence of Campylobacter in infants.

Another objective of this longitudinal study was to identify Campylobacter reservoirs within the selected households potentially associated with the early transmission of Campylobacter to infants. As observed for the infant stool samples, Campylobacter was highly prevalent within the households (>83%), especially livestock (cattle, chicken, goat, and sheep feces) and family members (sibling and mother stool samples). Furthermore, the Campylobacter loads in the infant stool samples were correlated with the Campylobacter loads in the mother stool samples (*r*^2^ = 0.18). These results are consistent with the first social interactions that the infants have with their mothers during the first months of life (e.g., holding the infant and breastfeeding). The transmission of Campylobacter between mothers and infants has been reported previously (24, 49). Therefore, our findings highlight the potential role of infected mothers in the early transmission of Campylobacter to their infants. We do not exclude the possibility that mothers’ hands can also be contaminated by the environment, or infants may be directly exposed to the environment. Whole-genome analyses and metagenomic sequencing of the Campylobacter isolates recovered from the human stool samples collected in this longitudinal study will provide further evidence to assess whether the human-to-human transmission of Campylobacter between family members is a major factor associated with infection of infants in early life. It is important to mention that in this study, Campylobacter was highly prevalent in sibling and mother stool samples. Although we did not collect information related to the mothers’ or siblings’ general health, we collected data related to women’s empowerment and child nutrition. However, we note that the prevalence in siblings was similar to that in infants at ~1 year of age, suggesting that the infection is sustained. The impact of these factors on the prevalence of Campylobacter is being determined and will be communicated in our future publication.

Interestingly, livestock, including sheep and goats, harbored the highest Campylobacter prevalences and loads in the surveyed households from eastern Ethiopia, which is in contrast to studies conducted in Europe and the United States where chickens were the major reservoirs of thermophilic Campylobacter (50, 51). This difference might be due to the diversity of Campylobacter but also because studies conducted in developed countries rarely focus on nonthermophilic Campylobacter (52–54). Our formative study conducted in the same region showed that several nonthermophilic Campylobacter species (i.e., C. hyointestinalis [potentially “*Candidatus* Campylobacter infans”], RM6137, and RM12175) in addition to the thermotolerant species C. upsaliensis, C. jejuni, and C. coli were predominant in child stool samples (31). Despite the high prevalence and load of Campylobacter in livestock feces, no direct correlations with the infant stool sample data were detected. On the other hand, the loads of Campylobacter in livestock feces (especially chickens and cattle) were strongly correlated with the Campylobacter load in the soil collected outside and inside the house. For example, the Campylobacter load in chicken feces was positively correlated with the load in soil collected outside the house (*r*^2^ = 0.60), which in turn was positively correlated with the load in mother stool samples (*r*^2^ = 0.69). Also, as mentioned above, the Campylobacter load in the infant stool samples was correlated with the Campylobacter load in the mother stool samples (*r*^2^ = 0.18). Similarly, the Campylobacter load in cattle feces was positively correlated with the load recovered from the floor inside the house covered with a carpet or plastic layer (*r*^2^ = 0.63), which in turn was positively correlated with the load in the infant stool samples (*r*^2^ = 0.36). Altogether, our findings highlight that Campylobacter infection of infants may potentially occur through two distinct ways, both involving soil as an intermediate between livestock and humans (Fig. 4B). Previous studies demonstrated the potential for Campylobacter to be persistent in soil, which could be a key factor in the transmission of Campylobacter within a household (55–57). Nevertheless, we do not exclude the possibility that other transmission pathways not investigated in this study may contribute to the persistence and dissemination of Campylobacter within a household in eastern Ethiopia and, thus, to the early infection of infants by Campylobacter. Such pathways would be difficult to detect by correlation analyses, and additional studies to model the exposure of infants in our cohort by including behavioral data are ongoing (Chen et al., unpublished; Li et al., unpublished). These analyses will provide insights into the potential impacts of infant health, household practices, and other spatiotemporal factors on the prevalence and load of Campylobacter in infants and associated household contacts. Differences in Campylobacter prevalences between kebeles were also detected in this study (especially in drinking water). We hypothesize that either the source of the water or the practices used to collect or store the drinking water in these households might enhance the introduction and persistence of Campylobacter in drinking water.

As we have described in a recently published study protocol (29), significant limitations were faced during the execution of this project. First and foremost, this longitudinal study was conducted during the coronavirus disease 2019 (COVID-19) pandemic. Furthermore, during this period, Ethiopia went through several unstable episodes associated with armed conflict and national elections. Together, these factors put the studied population under a great deal of stress and significantly disrupted their lives (e.g., drought, recurrent outbreaks in humans and livestock, and limited access to food and fuel), which may have affected the Campylobacter burden in eastern Ethiopia (29). In addition, limitations associated with sample collection were recorded. Limited quantities of stool samples (<1 g) could be collected from the studied infants during their first few months of life, and hesitation from the mothers to provide samples was encountered due to stigma, rumors, and cultural norms. Similarly, a limited amount of drinking water (i.e., 1 L per household per time point) could be collected from the selected households due to the already limited resources available and cultural norms. Thereby, despite the implementation of filtration steps to concentrate the samples using nylon vacuum filters, we acknowledge that our study might have underestimated the prevalence of Campylobacter in drinking water due to the limited amount of water available for the DNA extraction protocol. Due to the COVID-19 pandemic, certain supplies required for DNA extraction in Ethiopia were delayed, and thus, samples collected during the early phase of the project had to be stored at −80°C. Important differences in DNA extraction efficacies were detected between stool samples depending on the collection date, sample type, and storage conditions prior to DNA extraction. Briefly, we observed that if the raw samples (e.g., stool or fecal samples) were stored at −80°C for an extended period of time (e.g., several weeks) before performing DNA extraction, the DNA obtained was often of low yield (<50 ng/μL) and poor quality (260/230 ratio of <1.0; 260/280 ratio of <1.6), while DNA extracted from fresh samples (processed within a week after storage at −80°C) harbored high yields (>50 ng/μL) and good quality (260/230 ratio of >1.0; 2.0 > 260/280 ratio of >1.5; indicators of purity of the extracted DNA). Furthermore, DNA extraction efficacy issues were encountered depending on the consistency of the infant stool samples, which could be associated with the diet provided to the infants. Similar issues were encountered with chicken feces, which were associated with the presence of organic acids. Therefore, “log genome copies per 50 ng of DNA tested” are used for the interpretation of the Campylobacter load data in this study.

## MATERIALS AND METHODS

### Study area.

A longitudinal study involving 106 infants was conducted from December 2020 to June 2022. Participants were selected randomly from the 10 rural kebeles (synonymous with villages) of Haramaya woreda (synonymous with district), East Hararghe Zone, Oromia Region, Ethiopia. Haramaya woreda is located 500 km from Addis Ababa, the capital city of Ethiopia. The altitude of this woreda ranges from 1,400 to 2,340 m above sea level. It is named after the administrative center, Haramaya Town, and is home to Haramaya University. It is bordered on the south by Kurfa Chale, on the west by Kersa, on the north by Dire Dawa, on the east by Kombolcha, and on the southeast by the Harari Region. Haramaya woreda has 36 rural kebeles (the smallest administrative unit in Ethiopia) and three urban kebeles. The 2007 national census reported a total population for this woreda of 271,018, of whom 138,282 were men and 132,736 were women. A survey of the land in Haramaya woreda showed that 36.1% is arable or cultivable, 2.3% is pasture, 1.5% is forest, and the remaining 60.1% is considered built up, degraded, or otherwise unusable. Khat, vegetables, and fruits represent the major cash crops of this district (Haramaya District Agriculture and Livestock Office, unpublished data).

### Study design, sample size, sample collection, and interviews.

In rural Haramaya woreda, most of the houses are constructed with locally available materials (e.g., wood, mud, and aluminum layers). In most cases, these houses are composed of one room subdivided into stations based on the activity performed (e.g., sleeping, cooking, eating, and cleaning) and may have a door to control the flow between inside and outside. Based on the activity performed (especially sleeping and eating), the soil may be covered with a carpet or sheet to avoid direct contact with dust and dirt. In addition, the houses lack basic hygiene facilities (e.g., a latrine and a handwashing station). Thereby, dry and liquid wastes are commonly disposed of at locations a short distance from the house and dumped in the back or in front of the house. Each household also has a wide variety of livestock (chickens, cattle, sheep, and goats) living in proximity to the household. In some cases, at night, the livestock are brought inside the house, uncontained, to avoid issues with predators outdoors. Based on this information, human stool samples, livestock feces, and environmental samples were collected over time from each household to assess the prevalence and load of Campylobacter. A sample of 100 children allows the estimation of a 50% prevalence with a precision of 10% at a 95% confidence interval and a power of 80%. Full details of the enrollment process were described in our previously reported protocol (29).

Infant stool samples (*n* = 1,073) were collected monthly, starting from enrollment (7 days after birth) until 376 days of age. The first morning stools produced by the child were collected into a sterile plastic sheet placed inside a diaper (at least 10 g; however, it is important to mention that limited stool samples were available during the first months after infant birth [sometimes <1 g of stool was collected per time point]). The diapers and plastic sheets were sterilized in the laboratory using UV light (12 h). Sterilization was conducted the day of or 1 day prior to distribution to the study participants. Infant stool samples were transferred to a 207-mL Whirl-Pak bag and transported on ice to the laboratory for processing. The mother was asked if the infant had diarrhea on the day of sampling or the day before and about the number of loose stools in the last 24 h.

Additional human stool samples (i.e., mothers and siblings), livestock feces (i.e., goats, cattle, sheep, and chickens), and environmental samples (i.e., soil and drinking water) were collected biannually (*n* = 1,648). Human stool samples were collected by direct defecation onto a sterile plastic sheet and then transferred using a sterile wooden spatula into a 207-mL Whirl-Pak bag. No data on diarrheal symptoms were collected.

Livestock feces (fresh droppings; at least 10 g) were collected from cattle (*n* = 200), sheep (*n* = 197), goats (*n* = 199), and chickens (*n* = 161) using aseptic techniques. Feces were individually transferred into a 207-mL Whirl-Pak bag. Three soil samples per household (*n* = 453) were collected inside (*n* = 2; 1 sample where the floor is covered by a carpet or plastic sheet and 1 sample where the floor is made of dust and dirt) and outside (*n* = 1; 1 sample collected in front of the house) using boot socks humified with 207 mL of 10% skim milk. Each soil sample was collected by performing 15 steps within the designated area (preferably a 3- by 5-m area where children have access, making sure to cover most of the area). The used boot socks were removed using aseptic techniques and transferred into the original Whirl-Pak bag humified with 10% skim milk. One liter of drinking water samples (*n* = 147) that was stored in a larger container in the household (e.g., bottles and jerricans) was aseptically collected into two 532-mL Whirl-Pak bags. All samples mentioned above were transported on ice to the laboratory for processing.

Short interviews were conducted on every occasion where child stool samples were collected and included, among others, questions to the mother about whether the child had diarrhea at the time of sample collection or the day before. If the mother answered affirmative, she was asked how many loose stools the child had passed in the previous 24 h. Further details on short and long interviews were described in the previous study by Havelaar et al. (29), and the results will be reported elsewhere.

### Sample processing.

Upon arrival at the laboratory, the weight of each sample was recorded. Stool and fecal aliquots (0.5 g per aliquot) were transferred into 1.5-mL centrifuge tubes and stored at −80°C for DNA extraction. Soil samples (boot socks) were resuspended in 25 mL of buffered peptone water (pH 7; BD Difco) and manually homogenized for 1 min. The supernatant was transferred to 50-mL tubes and centrifuged for 10 min at 4,500 rpm. The pellet was carefully resuspended in 1 mL of peptone water and stored at −80°C for DNA extraction. Drinking water samples were filtered using a 500-mL Corning disposable 0.22-μm nylon vacuum filter. The membrane was aseptically removed using a sterile blade and cut into 1-cm^2^ pieces using sterile forceps, and the membrane was transferred to a 15-mL tube containing 14 mL of peptone water and 15 to 20 3-mm glass beads and homogenized for 1 min to release the cells from the membrane. The supernatant was transferred to another 15-mL tube and centrifuged for 10 min at 4,500 rpm. The pellet was carefully resuspended in 1 mL of peptone water and stored at −80°C for DNA extraction.

### Extraction of genomic DNA.

A QIAamp PowerFecal Pro DNA kit (Qiagen, CA, USA) was used to extract genomic DNA from human stool samples and livestock feces, according to the manufacturer’s instructions. Similarly, a DNase PowerSoil Pro DNA kit (Qiagen, Hilden, Germany) was used to extract genomic DNA from soil and drinking water samples, according to the manufacturer’s instructions. Briefly, for both kits, 0.25 g or 0.25 mL of the sample was used for DNA extraction, as recommended by the manufacturer. The genomic DNA was resuspended in 100 μL of nuclease-free water (Qiagen, CA, USA). The quality and quantity of the DNA were analyzed using a UV5 Nano spectrophotometer (Mettler Toledo, Columbus, OH). DNAs with poor quality were cleaned using a Zymo (CA, USA) genomic DNA clean and concentrator kit. DNA samples were stored at −20°C until further use.

### Detection of Campylobacter using TaqMan real-time PCR.

The detection of Campylobacter spp. in the human stool samples, animal feces, soil samples, and drinking water samples was performed using TaqMan real-time PCR. Genus-specific primers targeting Campylobacter 16S rRNA were used (forward primer, GATGACACTTTTCGGAGCGTAA; reverse primer, GCTTGCACCCTCCGTATTA; probe, CGTGCCAGCAGCC-MGB) (38). Each reaction mixture (25-μL final volume) contained 12.5 μL of PrimeTime gene expression master mix (Integrated DNA Technologies, USA), 0.1 nmol of forward and reverse primers, 0.05 nmol of the probe, 50 ng of normalized DNA, and nuclease-free water (Qiagen, Hilden, Germany). Real-time PCR runs were conducted using QuantStudio 5 (Applied Biosystems, Waltham, MA). Genomic DNAs extracted from Campylobacter jejuni (ATCC 81-176) and Campylobacter upsaliensis (ATCC 49816) were used as positive controls. Nuclease-free water was used as a negative control (background noise; duplicated control). The following thermocycler conditions were used: 1 cycle at 95°C for 10 min and 45 cycles at 95°C for 15 s followed by 55°C for 60 s.

A cutoff *C_T_* value of 35 was used for the detection of Campylobacter in the human stool samples, animal feces, soil samples, and drinking water samples (average *C_T_* value of nuclease-free water − 2.5 × standard deviation). A cutoff *C_T_* value of 29 was used for the detection of Campylobacter in the soil samples due to the low *C_T_* values obtained from the control soil samples compared to the water controls. *C_T_* values from positive samples were converted into Campylobacter genome copies per 50 ng of DNA tested based on a standard curve obtained using a protocol similar to the one described above. DNA extracted from a cocktail of five Campylobacter species (C. jejuni 81-176, C. lari ATCC 43675, C. fetus ATCC 33293, *C. helveticus* ATCC 51209, and C. upsaliensis ATCC 49816) (1 volume of DNA per strain normalized to 50 ng/μL) was used for the standard curve using a determined concentration of DNA. The DNA concentration tested were converted into genome copy numbers using the following formula: genome copies = [concentration of DNA tested (ng) × 6.0221 × 10^23^]/[average mass of 1 bp of DNA (660 g/mol) × 10^9^] (https://www.idtdna.com/pages/education/decoded/article/calculations-converting-from-nanograms-to-copy-number). A linear regression analysis was used to determine the relationship between the *C_T_* values and the genome copy numbers obtained for the determined concentration of DNA tested. The following equation was obtained: log_10_ Campylobacter genome copies = 10.51 − 0.24 × *C_T_* value.

### Statistical analysis.

Statistical analyses were performed using JMP Pro 16 software (SAS Institute, Cary, NC, USA) and R v4.3.0 (http://www.r-project.org/index.html). The data generated in this project were stored in REDCap (https://www.project-redcap.org/) for safekeeping and exported to csv files for analysis. The normality of the real-time PCR data was determined using the Shapiro-Wilks test. Differences in Campylobacter prevalences and loads between sample types, collection dates, and seasons were tested using chi-squared and Wilcoxon tests, respectively. To account for repeated measurements, logistic regression models were fitted to data on the presence or absence of Campylobacter in infant stool samples with age using generalized estimation equations. The Campylobacter load as a function of age was analyzed using generalized linear mixed models. Analyses were done for all samples and separately for samples from children with or without diarrhea as reported by the mother using the WHO case definition “the passage of three or more loose or liquid stools per day” (https://www.who.int/news-room/fact-sheets/detail/diarrhoeal-disease). Unconditional logistic regression models for symptomatic and asymptomatic infections were then conditioned on the presence or absence of the genus. Prediction intervals of the conditional models were recalculated from the variance of the unconditional models as *V_pq_* = *V_p_*/*p*^2^ + *V_q_*/*q*^2^, where *V_pq_* is the variance of the product of two predicted values, *p* (genus-level model) and *q* (model for symptomatic or asymptomatic infections), and *V_p_* and *V_q_* are the variances of the predicted values.

Hierarchical clustering analysis was conducted to identify infants with similar Campylobacter prevalence profiles over time. A multivariate analysis was used to identify correlations in the loads of Campylobacter between sample types (Pearson correlation and linear regression reported for each pairwise comparison axis). An association study (also called market basket analysis [58]) (JMP Pro 16) was conducted to determine whether the prevalence of Campylobacter in a specific sample type within the household could be linked to higher contamination risks for the infant. A minimal confidence level of 95% and a lift (a measure of the effectiveness of a predictive model calculated as the ratio between the results obtained with and those obtained without the predictive model) of 4 were used to identify associations between sample types of statistical importance. A *P* value of 0.05 was used as the cutoff for all of the statistical tests mentioned above.

### Ethics statement.

Ethical approval was obtained from the University of Florida Internal Review Board (IRB201903141), the Haramaya University Institutional Health Research Ethics Committee (COHMS/1010/3796/20), and the Ethiopia National Research Ethics Review Committee (SM/14.1/1059/20). Written informed consent was obtained from all participating households (husbands and wives) using a form in the local language (Afan Oromo). Research findings will be disseminated to community stakeholders, including participants, through the existing community advisory board (CAB). Findings will be disseminated to scientific, academic, policy, and development stakeholders through conferences and peer-reviewed journals and through the Feed the Future Innovation Lab for Livestock Systems. The project website https://livestocklab.ifas.ufl.edu/projects/caged/ provides access to all results.

A community advisory board, including a representative of the community, religious leaders (imams), woreda and kebele administrations, woreda women’s and children’s affairs, woreda bureau of health and agriculture, kebele health, and agricultural extension workers, was established to guide the research team for a better understanding of local context and entry into the community and is regularly engaged in the research (29). Only the project manager at Haramaya University and the data manager at the University of Florida have access to personally identifiable information in the REDCap database. Any data shared among researchers within the project were deidentified and blinded. Materials and data transfer agreements ensure the confidentiality of data when exchanged with international partners and others.
