# Supplementary material for: Prevalence and Load of the Campylobacter Genus in Infants and Associated Household Contacts in Rural Eastern Ethiopia: a Longitudinal Study from the Campylobacter Genomics and Environmental Enteric Dysfunction (CAGED) Project
Source: Appl Environ Microbiol. 2023 Jun 13;89(7):e00424-23. doi: 10.1128/aem.00424-23 (PMC10370295; doi:10.1128/aem.00424-23)

Supplemental Table 1. Population size by sample type for the selected kebeles

Kebeles (n=10)	Household selected	Samples tested	Human stools			Livestock feces				Environment	
			Infant	Mother	Sibling	Cattle	Chicken	Sheep	Goat	Drinking water	Soil
Adele Walta	10	210	97	16	14	19	13	18	17	4	12
Amuma	11	295	112	12	15	20	15	20	22	19	60
Bachaqe	8	209	83	12	14	15	14	16	15	10	30
Biftu Geda	11	273	121	15	15	20	15	19	20	12	36
Damota	12	319	119	18	18	24	17	23	20	20	60
Gobe Challa	9	228	86	10	14	15	12	18	17	14	42
Ifa Oromia	10	256	96	11	12	20	17	19	20	13	48
Kuro	11	277	97	10	11	21	20	20	18	20	60
Negeya	12	319	122	12	14	24	19	24	24	20	60
Qerensa Dereba	12	331	140	20	20	22	19	22	24	16	48
**Total**	**106**	**2,717**	**1,073**	**136**	**147**	**200**	**161**	**197**	**199**	**148**	**456**

Supplemental Table 2. *Campylobacter* prevalence in the kebeles selected for the longitudinal study.

**Kebeles**	**Samples tested**	**Total prevalence**	**Human stools**			**Livestock feces**				**Environment**	
			**Infant (n=1,073)**	**Mother (n=136)**	**Sibling (n=147)**	**Cattle (n=200)**	**Chicken (n=161)**	**Sheep (n=197)**	**Goat (n=199)**	**Drinking water (n=148)**	**Soil (n=456)**
Adele Walta	210	81%	73%	88%	93%	100%	100%	100%	94%	25%	67%
Amuma	295	68%	54%	83%	93%	100%	100%	100%	95%	47%	62%
Bachaqe	209	70%	61%	92%	100%	100%	79%	94%	100%	30%	67%
Biftu Geda	273	71%	64%	80%	93%	100%	87%	100%	95%	67%	47%
Damota	319	69%	60%	94%	94%	100%	88%	100%	100%	5%	63%
Gobe Challa	228	66%	59%	50%	93%	100%	100%	100%	100%	79%	43%
Ifa Oromia	256	76%	66%	82%	92%	100%	100%	100%	100%	54%	71%
Kuro	277	66%	59%	80%	82%	100%	90%	100%	100%	50%	45%
Negeya	319	71%	65%	92%	71%	96%	95%	100%	100%	20%	62%
Qerensa Dereba	331	76%	74%	80%	100%	100%	95%	95%	100%	63%	58%
**Total**	**2,717**	**73.6%**	**63.8%**	**83.1%**	**91.8%**	**99.5%**	**93.2%**	**99.0%**	**98.5%**	**43.2%**	**57.9%**

Supplemental Figure 1. *Campylobacter* prevalence by individual infant selected for this study (n=106). The *Campylobacter* prevalence was calculated based on the age of the infant when the stool samples were collected. The color of the cells is proportional to the prevalence. Cells with an “X”: no data available.

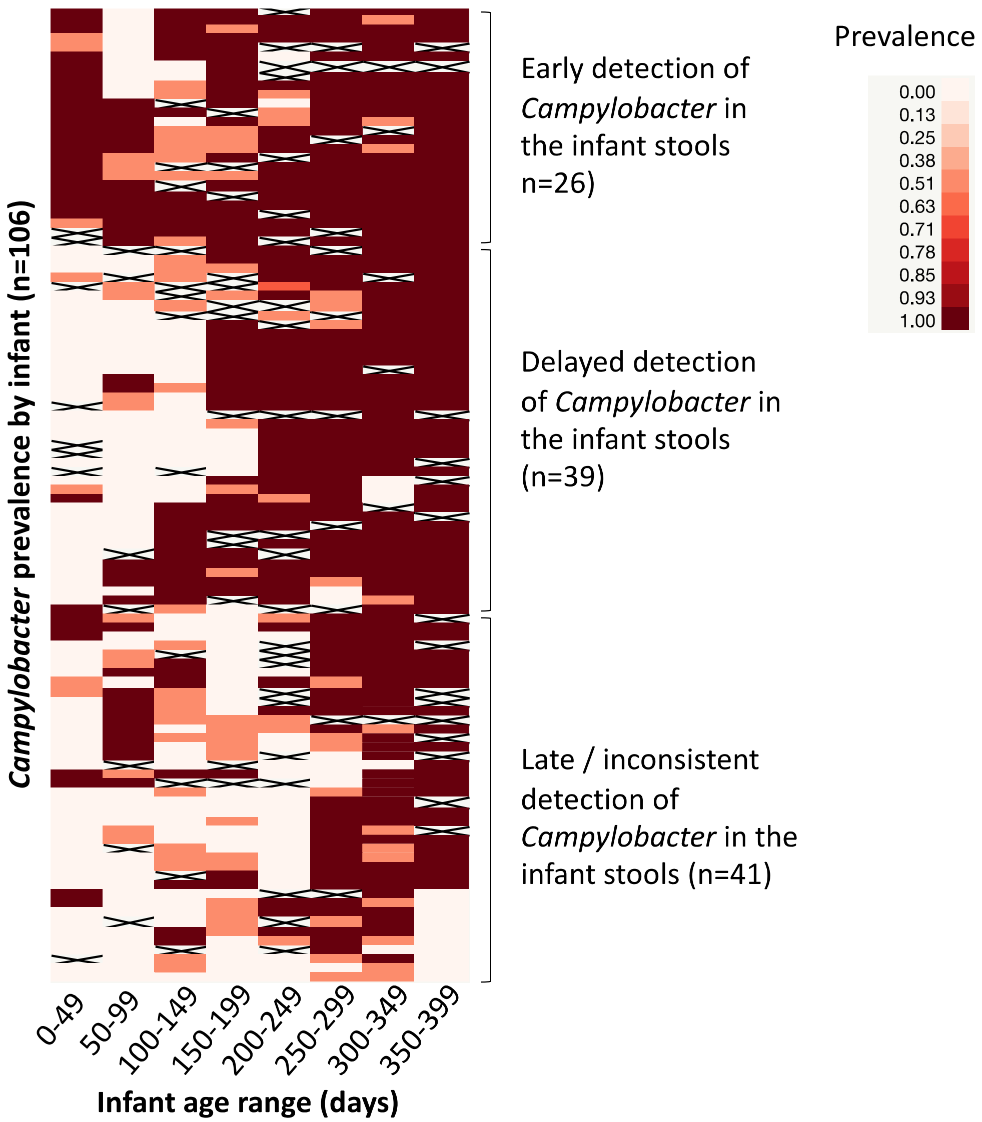

Supplemental Figure 2. Prevalence and abundance of *Campylobacter* in fields samples over time. *Campylobacter* prevalence (A) and load (B) data. Blue line and shade indicate the mean and 95% confidence interval. X axis indicates the collected data of the samples. The associated color indicates the seasons (rainy season in green from June to august and dry season in red from October to May). Brackets indicates Ramadan. “Infant”, “Mother” and “Sibling” are human stool data. “Cattle”, “Chicken”, “Goat” and “Sheep” are animal feces data. “Drinking water” and “Soil” are environmental data.

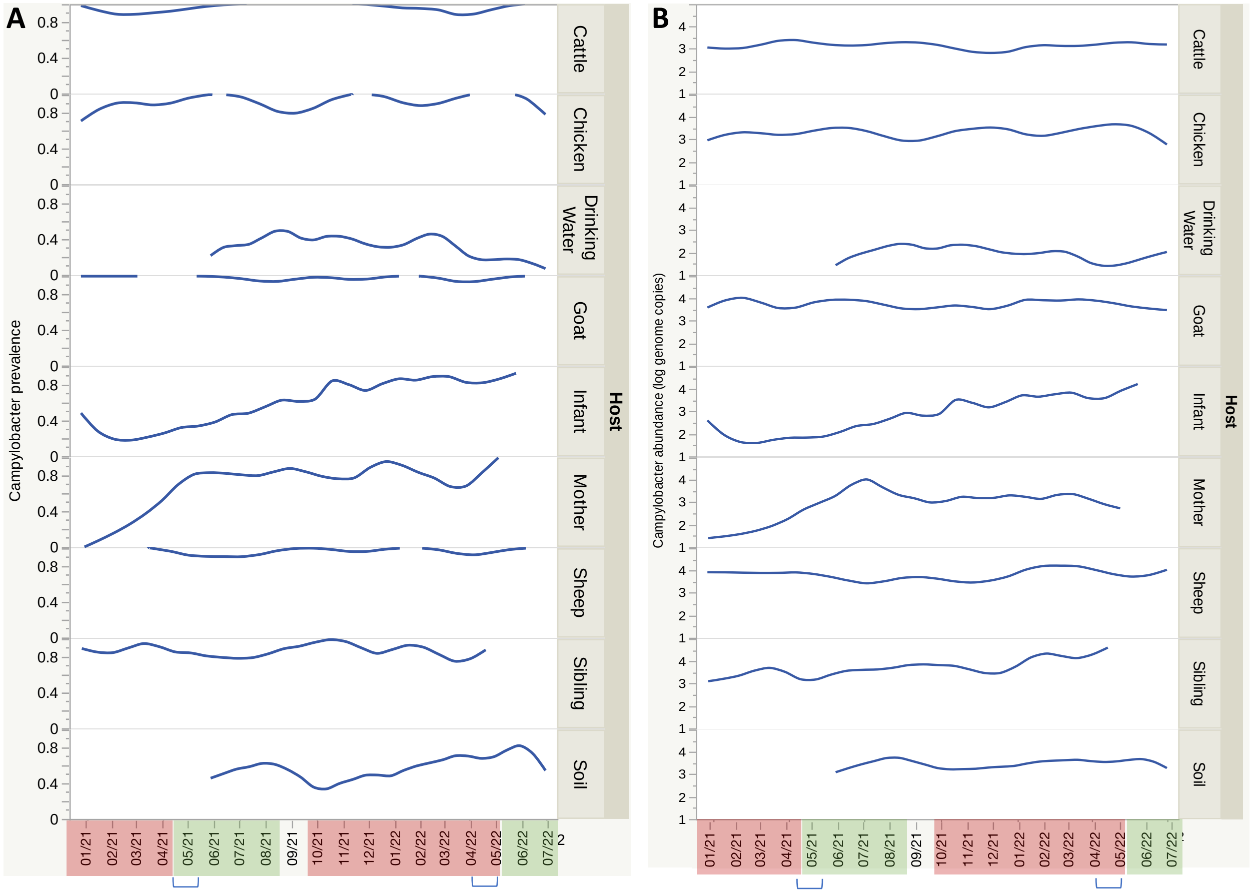

Supplemental Figure 3. Correlation in *Campylobacter* load between sample types by kebele. A) Overview of the correlation trends between samples types by kebele. For each kebele, *Campylobacter* load data were compared between samples types within the designated households. Only significant correlations ($P<0.05$) were used for the principal component analysis. Overall, the principal component analysis shows that different correlations in *Campylobacter* load between sample types were observed between kebeles (indicated by the orientation and length of the arrows). B) Details about the significant correlations in *Campylobacter* load between sample types by kebele. The colors in the heatmap are proportional with the intensity of the correlation (r^2 value; blue for negative and red for positive correlations) between the two sample types for the designated kebeles. The rows indicate the two samples type showing significant correlations ($P<0.05$). The columns represent each kebeles. The "X": no significant correlations detected. It is important to mention that the population size for some of the sample types by kebeles were small, which affected the resolution of the statistical analyses. Soil 0 indicates soil samples collected outside the house; Soil 1 indicates soil samples collected inside the house where the soil was covered with a carpet or plastic sheet; and Soil 2 indicates soil samples collected inside the house where the soil was not covered and likely close to the exit door.

A)

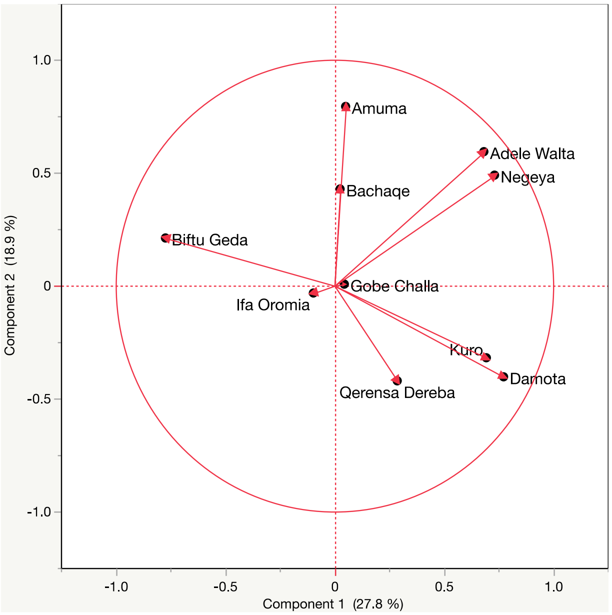

B)

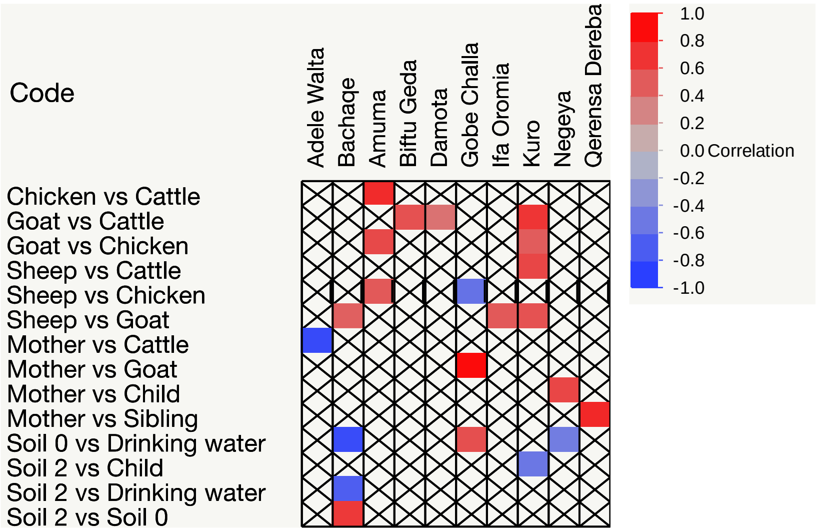

Supplement: Supplemental file 1 — Supplemental material. Download aem.00424-23-s0001.pdf, PDF file, 0.7 MB [file aem.00424-23-s0001.pdf]
